# Supplementary material for: Arabidopsis cryptochrome 2 forms photobodies with TCP22 under blue light and regulates the circadian clock
Source: Nat Commun. 2022 May 12;13:2631. doi: 10.1038/s41467-022-30231-9 (PMC9098493; doi:10.1038/s41467-022-30231-9)
Supplement: Supplementary file 1 — Supplementary Information [file 41467_2022_30231_MOESM1_ESM.pdf]

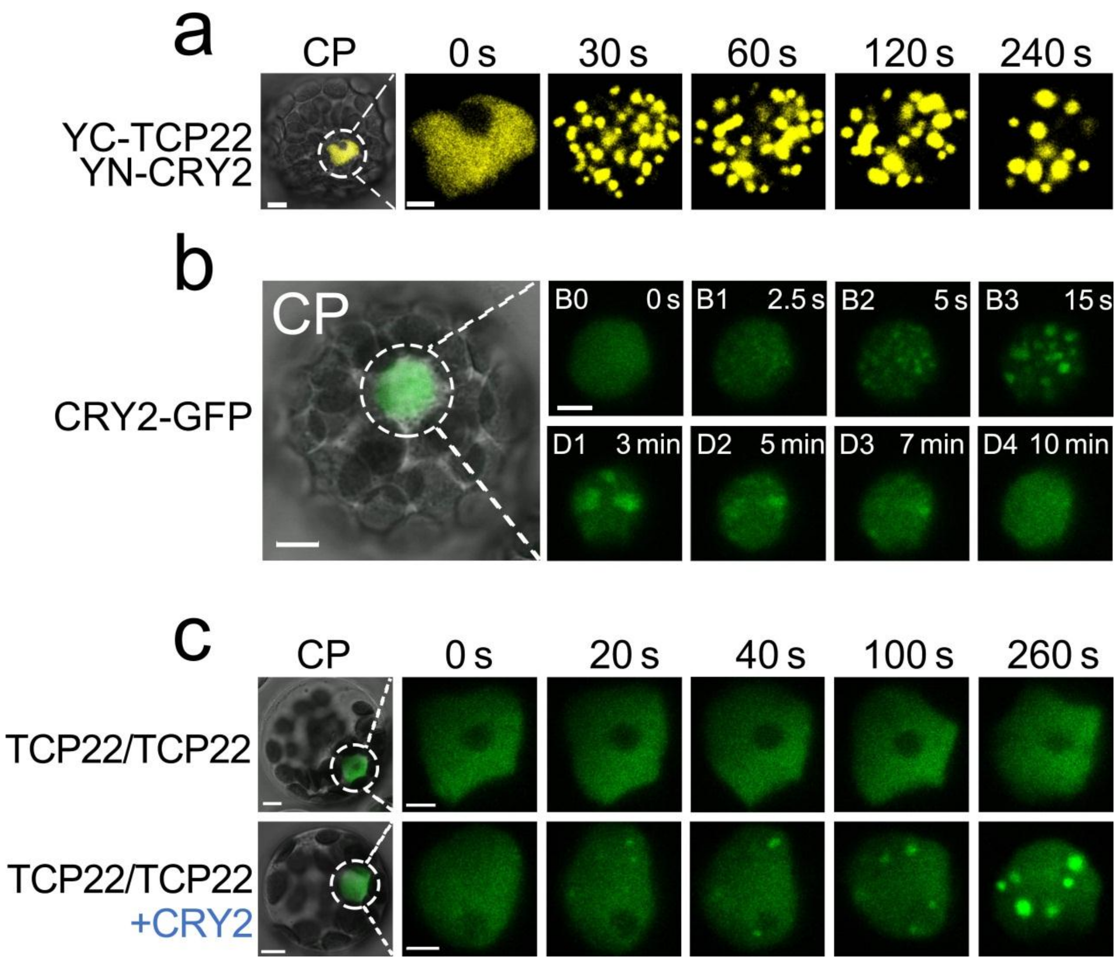

Supplementary Fig. 1 Photobody formation of TCP22 and CRY2 in protoplast.

a, Photobody formation of CRY2-TCP22 in 240 seconds (long exposure to blue light). Indicated plasmids were co-transfected into the protoplasts isolated from 3-week-old Arabidopsis (Col4). The florescence signal was captured using confocal microscopy with 514 nm laser. CP: Complete Protoplast, Scale bar, 5 μm (for the CP), 3 μm (for Nucleus). Similar results were observed in 5 different repeats. **b**, Blue light triggered CRY2-GFP to form the photobody *in vivo*. Dark incubated protoplasts expressing CRY2-GFP were activated by 488 nm laser at the indicated time. CP: Complete Protoplast. Scale bar, 5 μm (for the CP), 3 μm (for Nucleus). Three independent experiments show similar results. **c**, CRY2 induced TCP22 to form the photobody *in vivo*. Plasmids expressing NYFP-TCP22, CYFP-TCP22 and untagged CRY2 were co-transfected into the protoplasts isolated from 3-week-old Arabidopsis (*cry1cry2*). The fluorescence signals were captured at the indicated time points using the

continuous 488 nm laser. CP: Complete Protoplast. Scale bar, 5 μm for CP,3 μm (for Nucleus). Similar results were observed in 3 independent repeats.

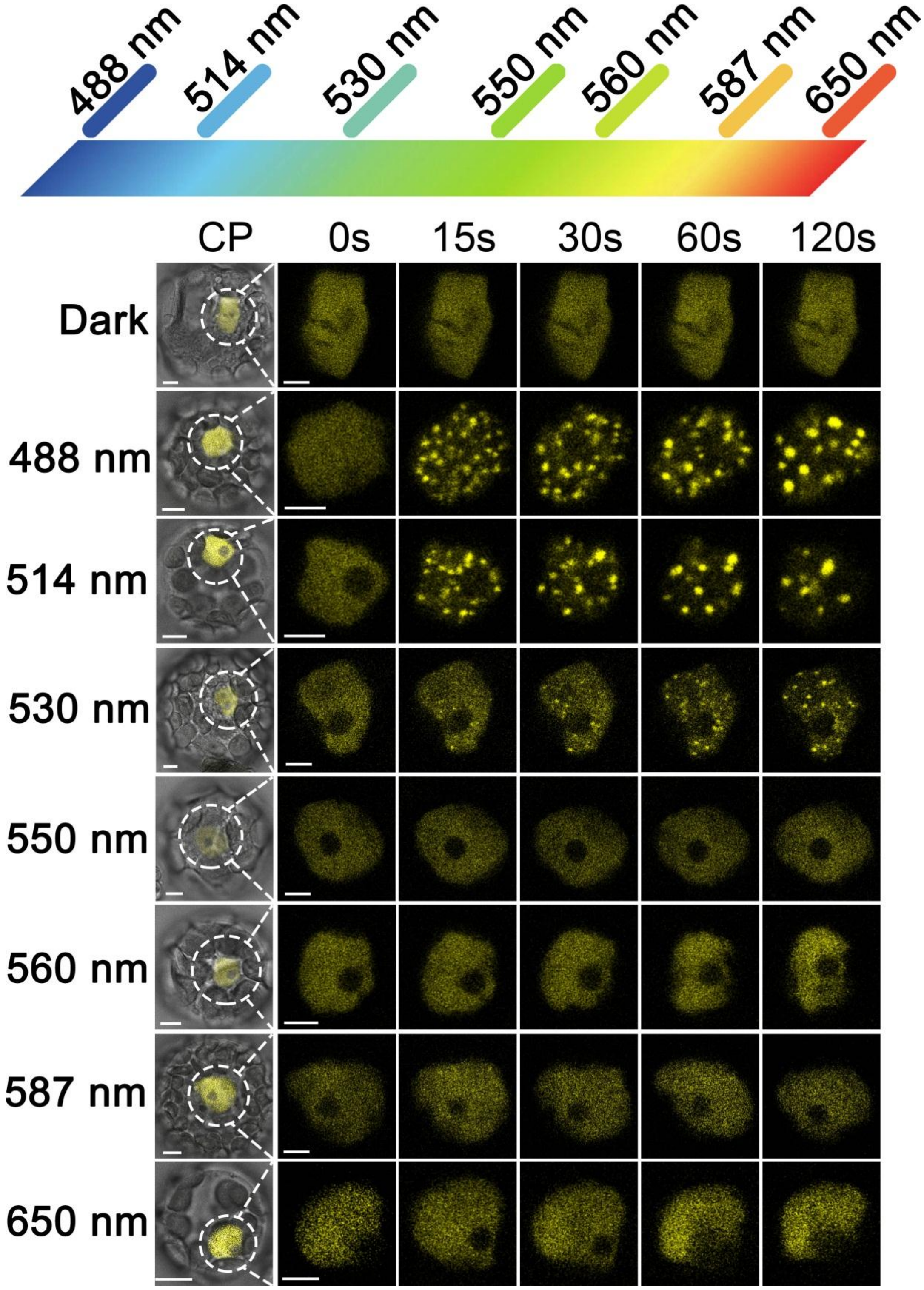

Supplementary Fig. 2 The wavelength specificity during CRY2-TCP22 photobody formation.

Protoplasts expressing NYFP-CRY2 and CYFP-TCP22 were exposed under dark environment, 488 nm, 514 nm, 530 nm, 550 nm, 560 nm, 587 nm and 650 nm lasers for the indicated time, respectively. Weak 514 nm laser with the shortest capturing time was used to capture the images of YFP. The weak 514 nm laser could not activate the photobody formation (see dark panel). CP: Complete Protoplast. Scale bar, 5 μm (for the CP), 3 μm (for Nucleus).

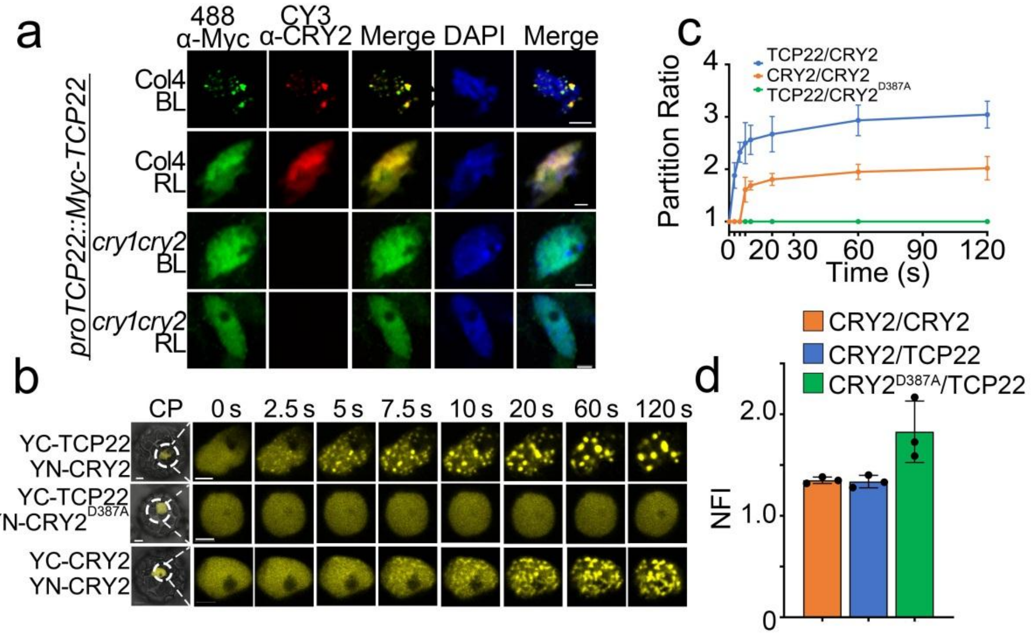

Supplementary Fig. 3 CRY2-dependent formation of CRY2-TCP22 photobodies.

a, Immunostaining of nucleus showed TCP22 formed the photobody in a CRY2 dependent manner under blue light. 10-day-old red light–grown Arabidopsis seedlings were treated with blue light (BL, 30 μmol m^{-2} s^{-1}) or kept in red light (RL, 30 μmol m^{-2} s^{-1}) for 30 min. Scale bar, 3 μm. **b**, Photobody formation of the indicated proteins in Col4 protoplasts under 514 nm laser. Scale bar, 5 μm for the CP (Complete protoplast), 3 μm for nucleus. Similar Results (n = 5 independent BiFC assays) were observed. **c**, The partition ratios of photobodies from n= 3 cells at the indicated time points corresponding to (**b**). Data are presented as mean ± SD. **d**, Normalized fluorescence intensity (NFI) of (**b**). The intensity of nuclei was calculated and normalized by the nucleus area. Data are presented as means ± SD (n =

3 cells in dark condition in b).

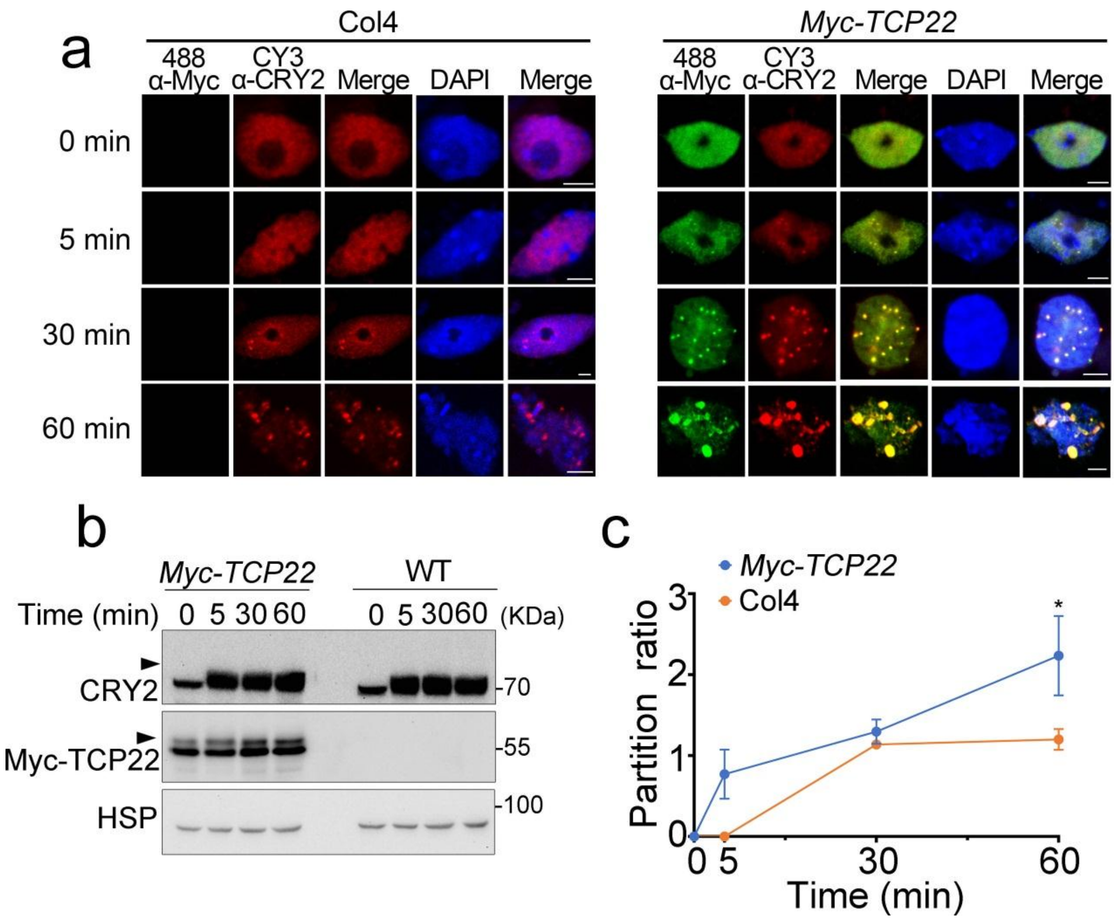

Supplementary Fig. 4 The morphology of CRY2 and CRY2-TCP22 photobody in Col-4, TCP22OX background under blue light.

a, Photobody formation of CRY2/TCP22 in the nuclei detected by the immunostaining assay in *Myc-TCP22*/Col4 and Col4 plants irritated by the indicated time under blue light (30 μmol m^{-2} s^{-1}). **b**, Immunoblots of samples prepared in (**a**). Arrowheads indicated phosphorylated TCP22 and CRY2. **c**, Partition ratio analysis of (**a**). Data are presented as mean ± SD. (n = 3 nuclei). Significance determined by two tailed student's *t* test (*p <0.05, p=0.024).

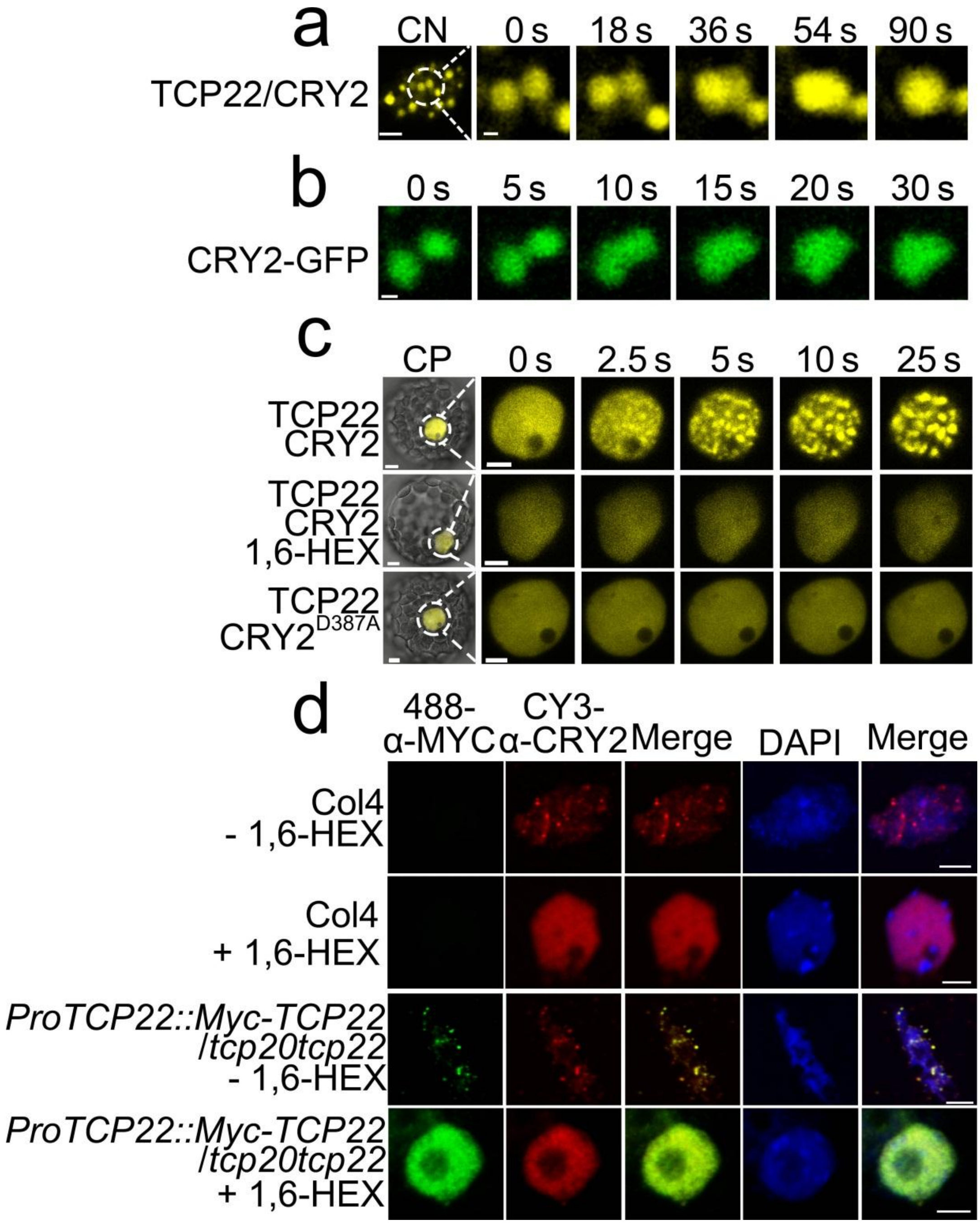

Supplementary Fig. 5 The photobody of CRY2 and CRY2/TCP22 sensitive to 1, 6-hexanediol

a, Fusion of CRY2-TCP22 photobodies under 514 nm laser in protoplasts. Plasmids expressing NYFP-CRY2 and CYFP-TCP22 were co-transfected into protoplasts isolated from 3-week-old Arabidopsis (Col4). CN: Cell Nucleus. Scale bar, 3 μm for Nucleus, 0.5 μm for photobodies. Similar results were observed in 3 independent repeats. **b**, Fusion of CRY2-GFP photobodies, Dark incubated protoplasts expressing

CRY2-GFP were activated by 488 nm laser, Scale bar,3 μm. **c**, 1,6-Hexanediol inhibited the formation of CRY2-TCP22 photobodies. Protoplast expressing NYFP-CRY2 or NYFP-CRY2^{D387A} and CYFP-TCP22 were added to W5 solution or W5 with 5% 1,6-Hexanediol for 5 min. The 514 nm laser was applied continuously to activate phase separation. Similar results were observed in 3 independent repeats. CP: Complete Protoplast. Scale bar, 5 μm (for the CP), 3 μm (for Nucleus). **d**, Immunostaining of nucleus showed the photobody formation in both WT and *ProTCP22::Myc-TCP22*/*tcp20tcp22* was inhibited by 1,6-Hexanediol. 10-day-old red light-grown Arabidopsis seedlings were treated with or without 10% (w/v) 1,6-Hexanediol for 30 min and then transferred to blue light (30 μmol m^{-2} s^{-1}) for 30 min. Scale bar, 3 μm.

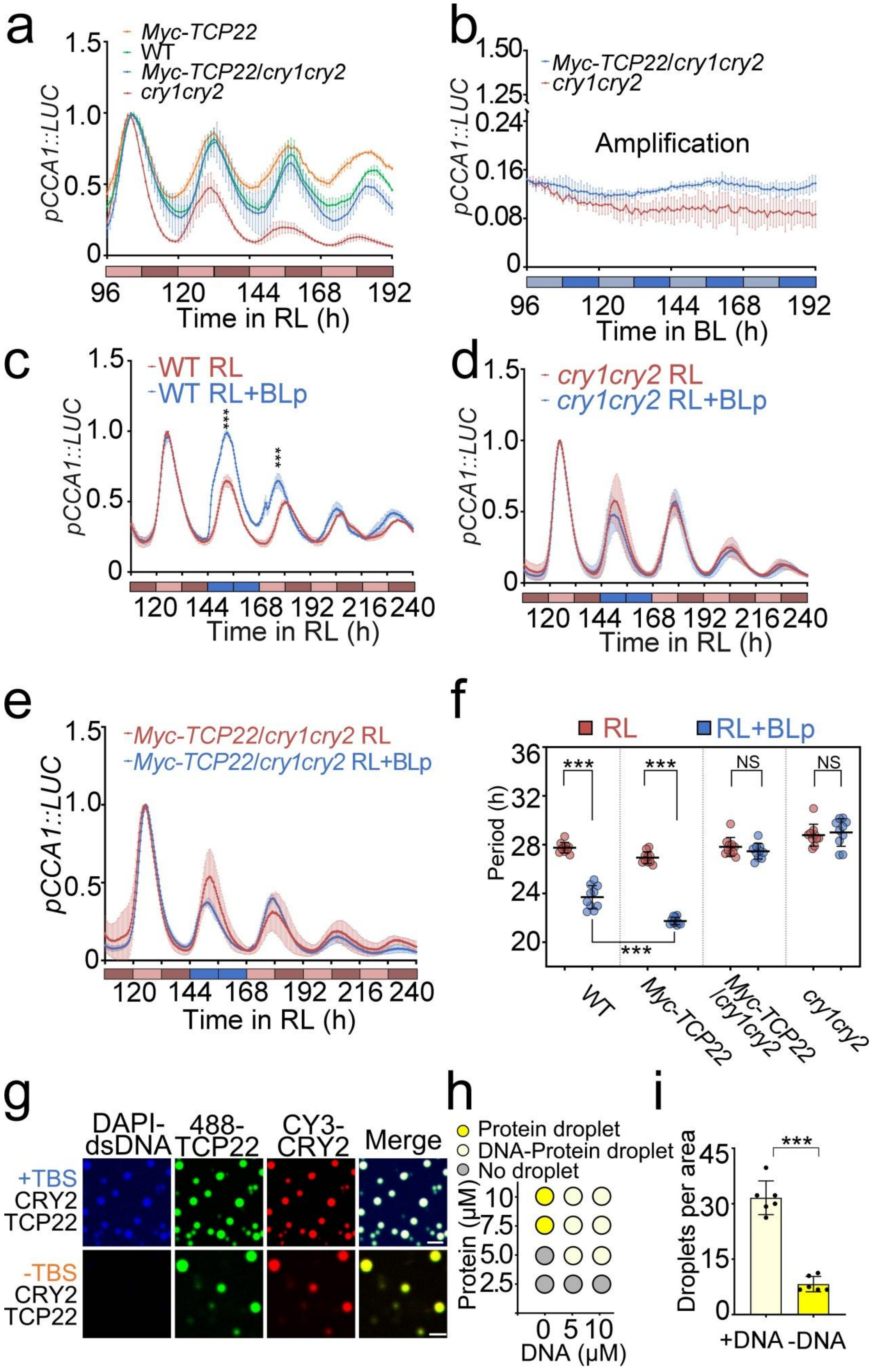

Supplementary Fig. 6 Controls related to Figure. 3 and TBS motif

enhancing the formation of CRY2-TCP22 droplet .

a, Bioluminescence analysis of *pCCA1::LUC* expression in genotypes of WT, *Myc-TCP22*/WT, *cry1cry2* and *Myc-TCP22*/*cry1cry2* under continuous red light treatment. Seedlings were synchronized under 12 h light/12 h dark light cycle (75 μmol m^{-2} s^{-1}) for 3 days in a 96-well plate and then transformed into continuous red light (5 μmol m^{-2} s^{-1}) for 4 days for training. The bioluminescence signal of each sample was detected in the following time with 1 h intervals. Data are presented as mean ± SD (n = 3 independent assays). **b**, Bioluminescence analysis of *pCCA1::LUC* expression in *cry1cry2*, *Myc-TCP22*/*cry1cry2* retained and highlighted from Fig. 3c.**c-d**, and Blue light pulse assay showed the expression and rhythm change of *pCCA1::LUC* in WT (**c**) and *cry1cry2* (**d**), respectively. The method was described above in Fig. 3d. Data are presented as means ± SD (n = 3 independent assays). Significance determined by two tailed student's *t* test (***p < 0.001, p=1.95x10^{-4}, 4.12x10^{-4} respectively). **e**, Blue light pulse assay in *Myc-TCP22*/*cry1cry2*, 12 h light/12 h dark grown plants were transferred to continuous red light (RL, 10μmol m^{-2} s^{-1}) except a blue light pulse (BLp, 15 μmol m^{-2} s^{-1}). Data are presented as mean ± SD (n = 3 independent assays). Significance determined by two tailed student's *t* test (***p <0.001, p=3.53x10^{-10}, 7.66x10^{-6}, 1.82x10^{-16}, 0.259, 0.633, respectively, NS, No significance). **f**, *CCA1* periods of the indicated genotype lines were calculated by FFT-NLLS algorithm in various light conditions, Data are presented as mean ± SD (n=10 independent assays). **g**, Photobody formation of iFlour-488-labeled TCP22 (8 μM) and CY3 labeled CRY2 (2 μM) were mixed with the DNA fragments (20 μM) from the TBS region in *CCA1* (DAPI-labeled) or not. Scale bar, 2.5 μm. **h**, Phase diagram of DNA and proteins mixture in different concentrations. CRY2 and TCP22 proteins were mixed in a 1:4 molar ratio. Three similar results were observed. **i**, Droplets per area in the presence or absence of DNA were shown using data from (**g**). Data are presented as mean ± SD, n=6 visual field droplets for each sample were calculated. Two tailed student's *t* test, ***p < 0.001, p=4.58x10^{-7}.

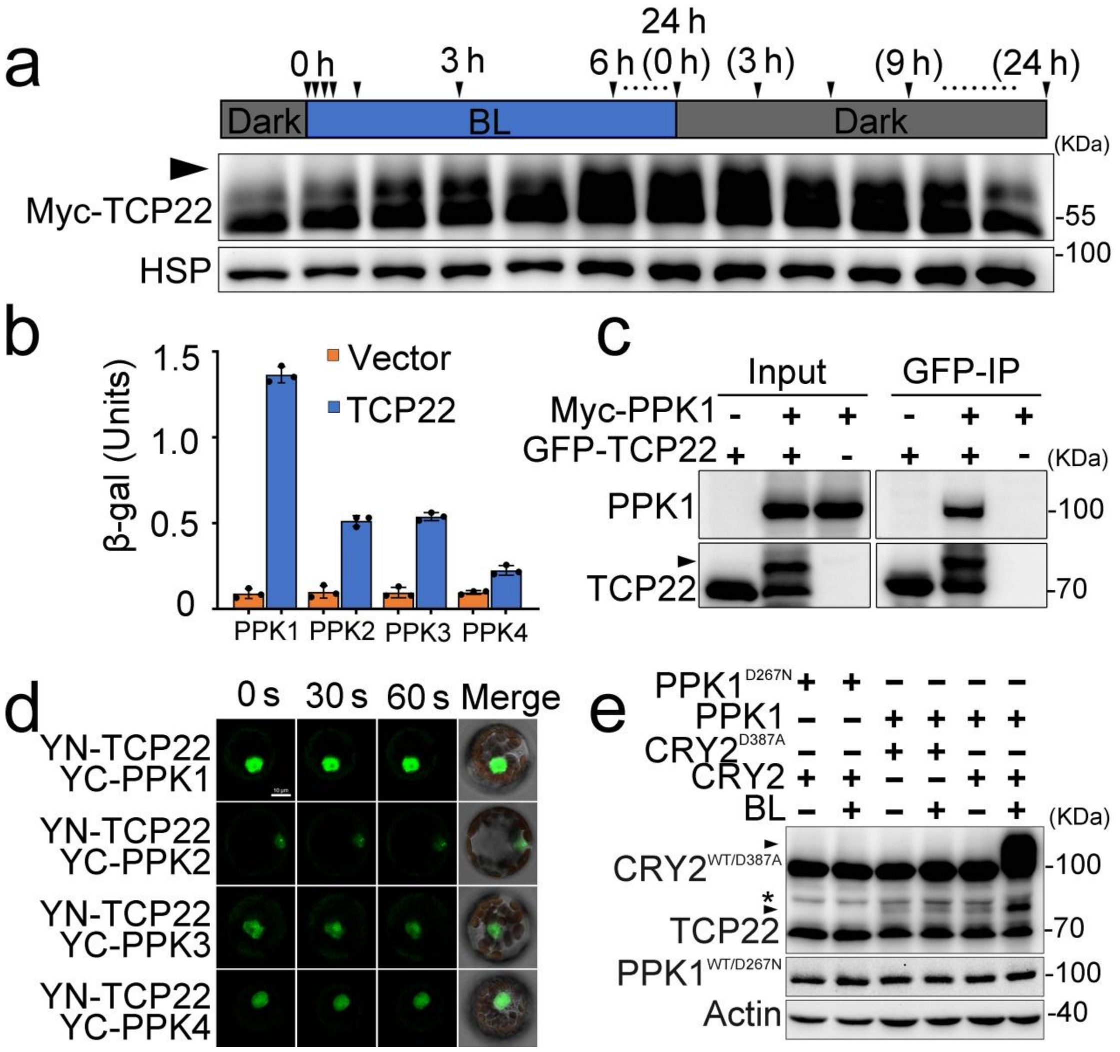

Supplementary Fig. 7 PPK1 interacts with and phosphorylates TCP22 *in vitro* and *in vivo*.

a, Blue light promoted TCP22 phosphorylation *in vivo*. The 6-day-old etiolated seedlings expressing *35S:: Myc-TCP22* were exposed to blue light (45 µmol m^{-2} s^{-1}) for the indicated time and collected for the immunoblot. Myc-antibody (TCP22) and HSP-antibody (loading control) were probed for each sample. Black triangles in the bar graph represented the time points correspondent to the immune-blot picture below. BL: 0 s, 5 min, 15 min, 30 min, 1 h, 3 h, 6 h, 24 h; Dark: 0 h, 3 h, 6 h, 9 h, 24 h. The arrowhead indicated the phosphorylated TCP22. **b**, Liquid assay of yeast two hybrid demonstrated the interaction between TCP22 and PPKs. Yeast cells expressing PPKs-BD were used as bait and TCP22-AD (blue bar) or empty vector as prey (orange bar). Data are presented as mean ± SD (n = 3 technical repeats). **c**, PPK1

interacted with TCP22 and catalyzed phosphorylation of TCP22 shown by Co-IP. HEK-293T cells co-expressing GFP-TCP22 and Myc-PPK1 were analyzed by immunoblots probed with the anti-Myc (PPK1) or anti-GFP (TCP22), respectively. Two independent experiments show similar results. **d**, BiFC assay of TCP22 and PPKs. Plasmids carrying the indicated gene were co-transfected into protoplasts isolated from 3-week-old Arabidopsis Col4. The images were captured using confocal microcopy with 514 nm laser. Similar results were observed in three independent repeats. Scale bar, 10 μm. **e**, CRY2 induced phosphorylation of TCP22 catalyzed by PPK1 in response to blue light. HEK-293T cells co-expressing the indicated proteins were irradiated by blue light (30 μmol m^{-2} s^{-1}) or kept in dark. The total protein was isolated and analyzed by immunoblots probed with the anti-Myc (PPK1, $PPK1^{D267N}$) and anti-GFP (CRY2, $CRY2^{D387A}$, TCP22) respectively. Actin was used as a loading control. Arrowheads indicated the phosphorylated TCP22 and CRY2. Asterisk indicated non-specific band. Two independent experiments show similar results.

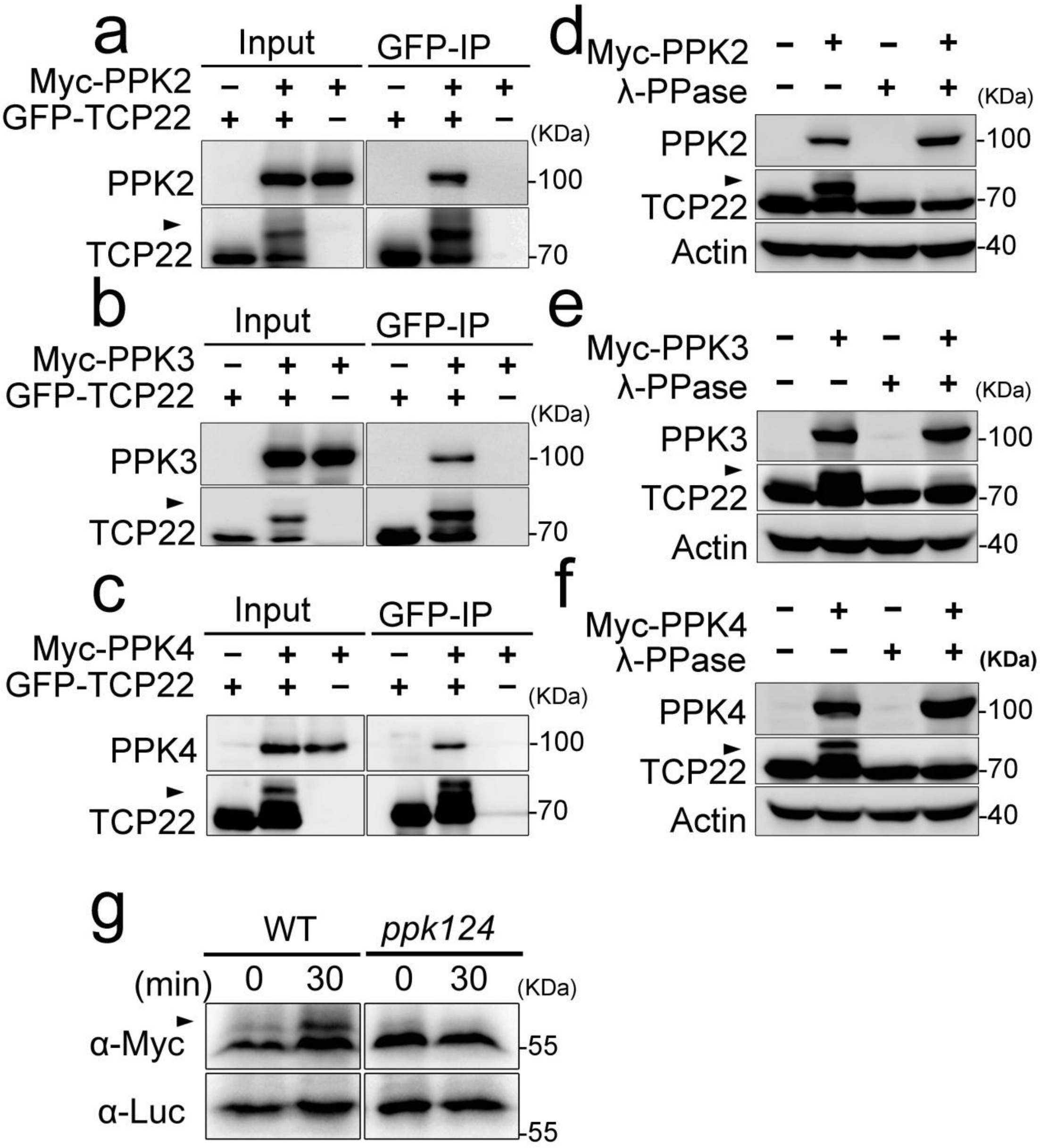

Supplementary Fig.8 PPK2, PPK3 and PPK4 interact with and phosphorylate TCP22, respectively.

a, **b**, **c**, TCP22 interacted with PPK2 (**a**), PPK3 (**b**), PPK4 (**c**) in HEK-293T cells as shown by Co-IP. HEK-293T cells were co-transfected with GFP-TCP22 and Myc-PPKs. After 36 h incubation, the total protein was isolated and analyzed by immunoblots probed with the anti-Myc (PPKs) or anti-GFP (TCP22). Arrowheads indicated phosphorylated TCP22. **d**, **e**, **f,** Phosphorylation of TCP22 catalyzed by PPK2 (**d**), PPK3 (**e**), PPK4 (**f**) in HEK-293T cells shown by immunoblots assays.

HEK-293T cells were co-transfected with GFP-TCP22 and Myc-PPKs. After incubation for 36 h, the total protein was isolated and treated with λ-PPase (+) or without λ-PPase (-). Samples were analyzed by immunoblots probed with the anti-Myc (PPKs) or anti-GFP (TCP22) antibody. Actin was used as a loading control. Arrowheads indicated phosphorylated TCP22. **g**, TCP22 phosphorylation in the absence of PPKs (*ppk124*) or presence (WT) responding to the blue light (30 μmol m^{-2} s^{-1}) shown by immunoblots assays. Four-day-old Arabidopsis including WT, and *ppk124* seedlings were infected with Agrobacterium C58C1 (pTiB6S3ΔT)^H carrying the vector pDT1 *ACTIN2::4×Myc-TCP22 UBQ10::LUC* for 3 days in a 16-h/8-h light/dark cycle (75 μmol m^{-2} s^{-1}). LUC was used as the transformation level of the vector. Arrowheads indicated phosphorylated TCP22. The above experiments were repeated at least two times with similar results.

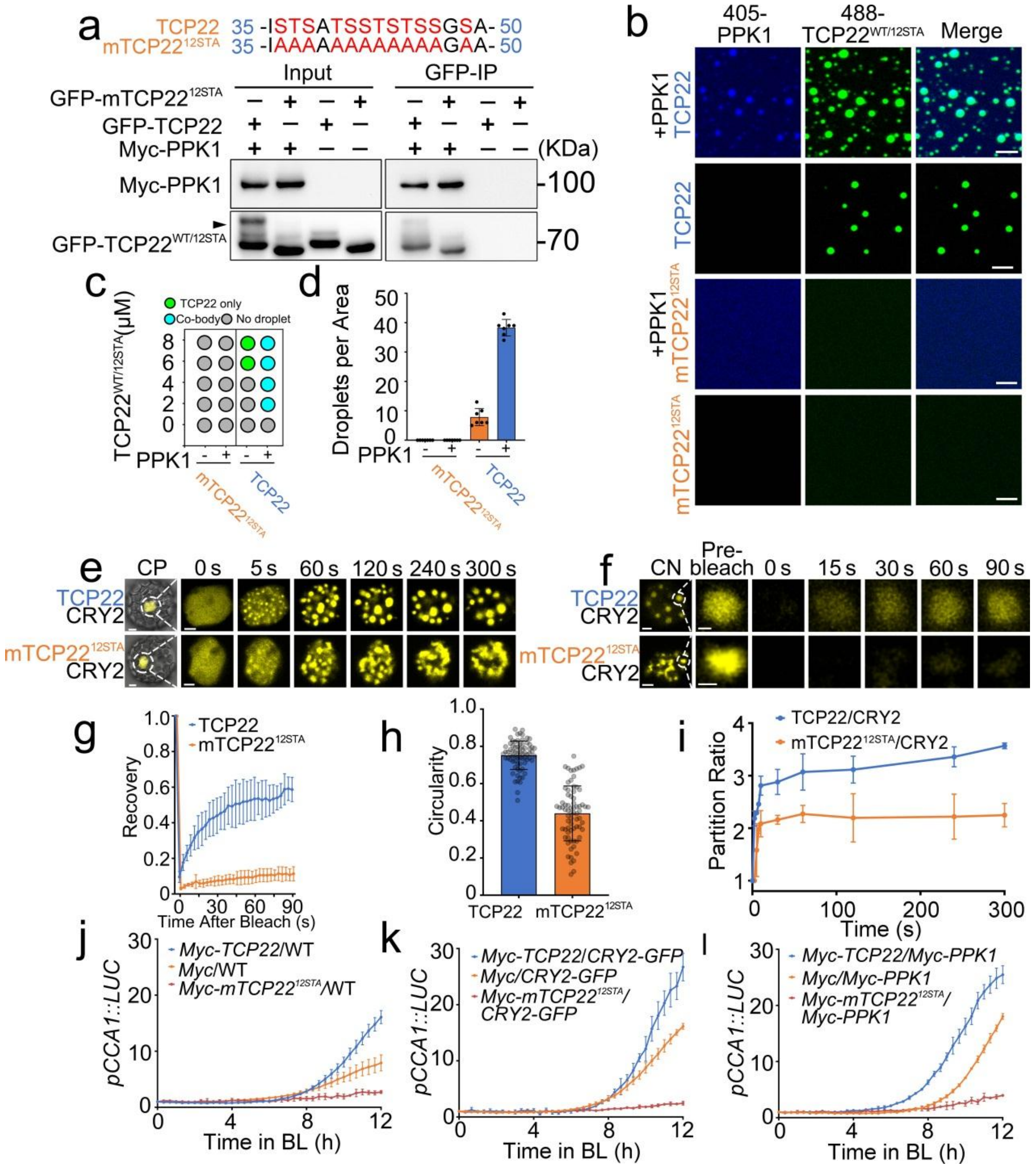

Supplementary Fig. 9 Inability of mutant TCP22^{12STA} on being phosphorylated, forming photobody and promoting the photobody formation of CRY2.

a, PPK1 was not able to phosphorylate mTCP22^{12STA} as shown by Co-IP assay (bottom). The input and IP products were analyzed by the immunoblot, probed by anti-GFP (TCP22 or mTCP22^{12STA}) or anti-Myc (PPK1). Arrowheads indicated phosphorylated TCP22. The mutation sequence (RED) of TCP22 was displayed (top). Two independent experiments show similar results. **b**, PPK1 enhanced the formation

of TCP22 droplets but not mTCP22^{12STA} *in vitro*. Purified 8 μM His-TCP22 or mTCP22^{12STA} and 5 μM His-PPK1 were labeled with iFluor 488 or iFluor 405. BSA with the same concentration of PPK1 were added as a negative control (-PPK1). The droplets were observed by a confocal microscopy with 405 nm and 488 nm lasers. Similar results were observed in independent repeats. Scale bar, 2 μm. **c**, Phase diagram indicated the droplets state of TCP22 or mTCP22^{12STA} and PPK1-TCP22 mixture in different protein concentrations. Grey cycle indicated no droplets were observed. Green and blue cycles represented the droplets of TCP22 or PPK1-TCP22 mixtures observed at the indicated protein concentrations, respectively. **d**, Quantification of droplets with or without PPK1. Data are presented as means ± SD (n= 7 different images captured by confocal microscope under the condition of B), the droplet number of each capture image were calculated, and the average number of droplets was indicated as droplets per area. **e**, Photobody formation of mTCP22^{12STA} and CRY2 *in vivo*. Indicated plasmids were co-transfected into the protoplasts isolated from 3-week-old Arabidopsis (Col4). The fluorescence signal was captured by the confocal microscopy with the 514 nm laser. CP: Complete Protoplast. Scale bar, 5 μm for the CP, 2 μm for Nucleus. Similar results were observed in independent repeats (n = 3). **f**, FRAP assay for CRY2-TCP22 or CRY2-mTCP22^{12STA} photobodies in (**e**). Photobodies were bleached after being irradiated by blue light of 2 minutes. Similar results were observed in three independent repeats. Scale bar, 3 μm for Nucleus, 0.5 μm for photobodies. CN, Cell Nucleus. **g**, Quantification of FRAP as indicated in (**f**). Plot showing the time course of the recovery after photobleaching photobodies in (**f**). Data are presented as mean ± SD (n = 7 independent FRAP experiments). **h**, Circularity of the CRY2-TCP22 or CRY2-mTCP22^{12STA} photobodies in the assay same as (**e**). Data are presented as means ± SD (n=7 cells calculated for each sample after irradiation with blue light for 2 minutes). The circularity was calculated using the following formula, Circularity = 4π (photobody area) / (photobody perimeter)^2. **i**, Partition ratio of CRY2-TCP22 and CRY2-mTCP22^{12STA} photobodies in (e). After being irradiated by blue light for 2 min. The partition ratio was calculated by the photobody intensity divided by the nucleus mean intensity. The partition ratio of

nuclei that failed to form photobodies was set to 1. Data are presented as mean ± SD (n=3 cells). **j**, **k**, **l**, CRY2 and PPK1 enhanced *CCA1* expression via phosphorylated TCP22 but not mTCP22^{12STA} as shown by bioluminescence assay. Bioluminescence analysis of *pCCA1::LUC* expression in indicated genotypes using the Agrobest method. Seedlings were irritated by blue light (10 μmol m^{-2} s^{-1}) in the 96-well plate, Luciferase signals were collected at 20 min intervals. Data are presented as mean ± SD (n = 3 independent assays).

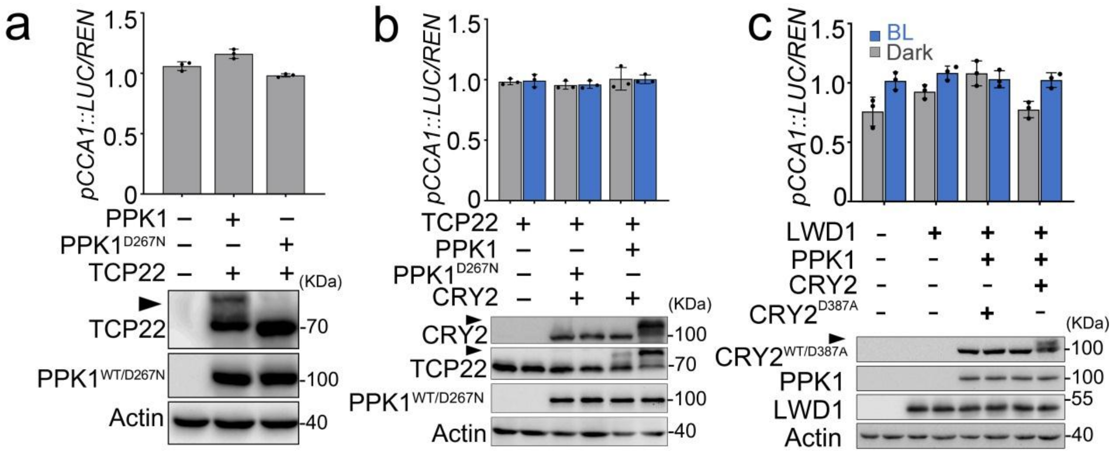

Supplementary Fig. 10 Both TCP22 and LWD1 are necessary for the transcriptional activation of *CCA1*.

a, TCP22 and PPK1 could not activate the expression of *CCA1* in the absence of LWDs as shown by the dual luciferase assay. *pCCA1::LUC* was co-expressed with the indicated proteins in HEK-293T cells. The relative luciferase (LUC) signal was normalized to the Renilla luciferase (REN) signal. Data are presented as mean ± SD (n = 3 independent assays). Expression levels of each protein in each sample were estimated by immunoblot. **b**, CRY2, TCP22, PPK1 could not activate the expression of *CCA1* in the absence of LWDs under blue light or dark treatment as shown by the dual luciferase assay. HEK-293T cells co-expressing the indicated proteins were irradiated with blue light (Blue columns, 30 μmol m^{-2} s^{-1}) or kept in dark (Grey columns) before the measurement of the LUC/REN signal. Data are presented as mean ± SD (n = 3 independent assays). Expression levels of each protein in each sample were estimated by immunoblot. **c**, CRY2, LWD1, PPK1 could not activate the expression of *CCA1* in the absence of TCP22 under blue light or dark treatment as

shown by the dual luciferase assay. Expression and analysis were the same as in (**b**), with the exception of indicated proteins. Data are presented as mean ± SD (n = 3 independent assays).

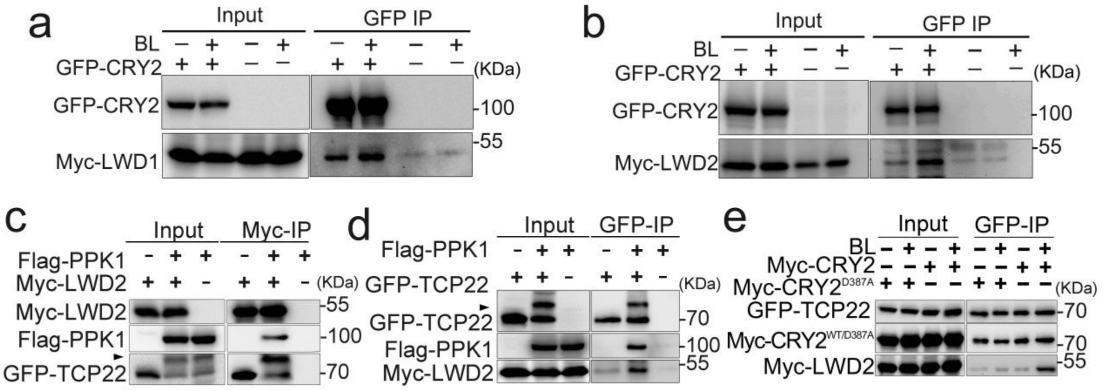

Supplementary Fig. 11 The interaction of LWD-TCP22 regulated by PPK1 and CRY2

a-b, Co-immunoprecipitation (Co-IP) assay showing the interaction between LWD1(**a**) or LWD2(**b**) and TCP22 in HEK-293T cells. The cells were treated with blue light (+ BL, 30 μmol m^{-2} s^{-1}) for 3 h or kept in the dark (-BL). The immunoprecipitation signals were probed by anti-GFP (CRY2) or anti-Myc (LWD1 or LWD2), respectively. **c**, LWD2 interacted more strongly with the phosphorylated TCP22 than with the unphosphorylated TCP22 as demonstrated by Co-IP assay. Indicated proteins were co-transfected into HEK-293T cells. Protein complex was immunoprecipitated with Myc-LWD2, and probed by anti-Myc (LWD2), anti-GFP (TCP22) and anti-Flag (PPK1), respectively. The arrowhead indicated the phosphorylated TCP22. **d**, PPK1 enhanced the interaction between LWD2 and TCP22 as demonstrated by Co-IP assay. The experiment and the conditions were the same as in (**c**), with the exception that the protein complex was immunoprecipitated by GFP-TCP22. The arrowhead indicated the phosphorylated TCP22. **e**, CRY2 enhanced the interaction between LWD2 and under blue light as demonstrated by Co-IP assay. HEK-293T cells co-expressing the indicated protein were irradiated by blue light (+BL, 30 μmol m^{-2} s^{-1}) or kept in dark (-BL). Protein complex was immunoprecipitated by GFP-TCP22, and probed by

anti-Myc (CRY2, CRY2^{D387A}, LWD2), anti-GFP (TCP22), respectively. The above experiments were repeated at least two times with similar results.

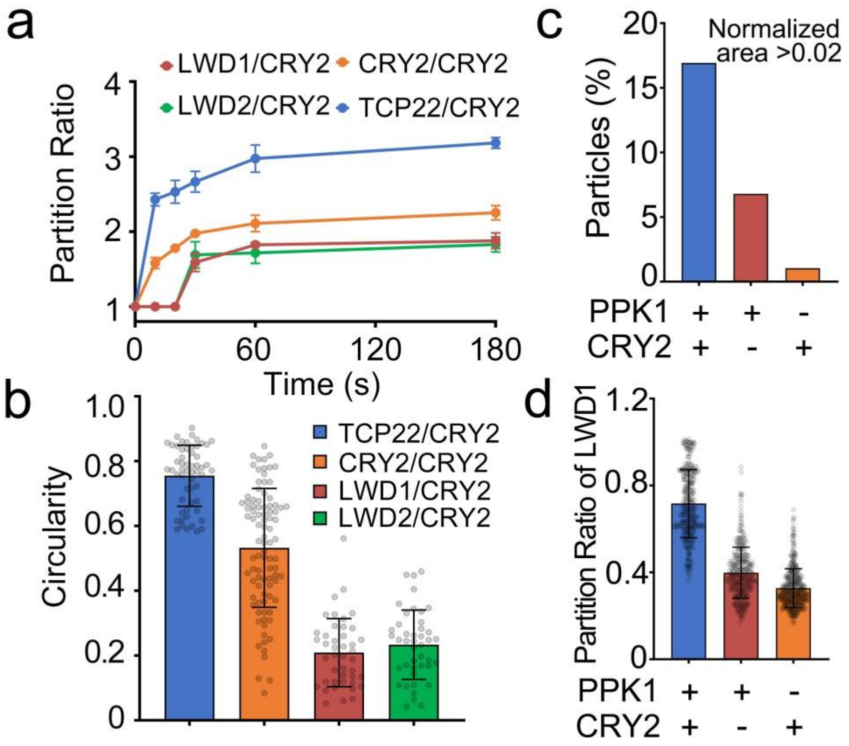

Supplementary Fig. 12 Statistical analysis of the partition ratios, circularity and size distribution of the indicated photobodies.

a, Partition ratios of CRY2-LWD1, CRY2-LWD2, CRY2-CRY2, and CRY2-TCP22 photobodies from n = 3 cells at the indicated time point. were calculated from 3 different repeats of the assay similar to Fig. 5d and Supplementary Fig. 3b after irradiation with blue light of the indicated time point respectively. The partition ratio was calculated by the photobody intensity divided by the nucleus mean intensity. The partition ratio of nuclei that failed to form photobodies was set to 1. Data are presented as mean ± SD. **b**, Circularity of the CRY2-LWD1, CRY2-LWD2, CRY2-CRY2, and CRY2-TCP22 photobodies. n = 6 cells were measured from Fig.

5d and Supplementary Fig. 3b after being irradiated by blue light of 3 min respectively. The circularity was calculated by following formula, Circularity = 4π (photobody area) / (photobody perimeter)^2. Data are presented as mean ± SD. **c**, Size distribution of droplets of Fig. 5h. n = 8 visual field droplets for first column and n = 6 for second and third column were calculated. **d**, Partition ratio of Fig. 5h. Data are presented as mean ± SD（n=5 visual field droplets for each sample were calculated).

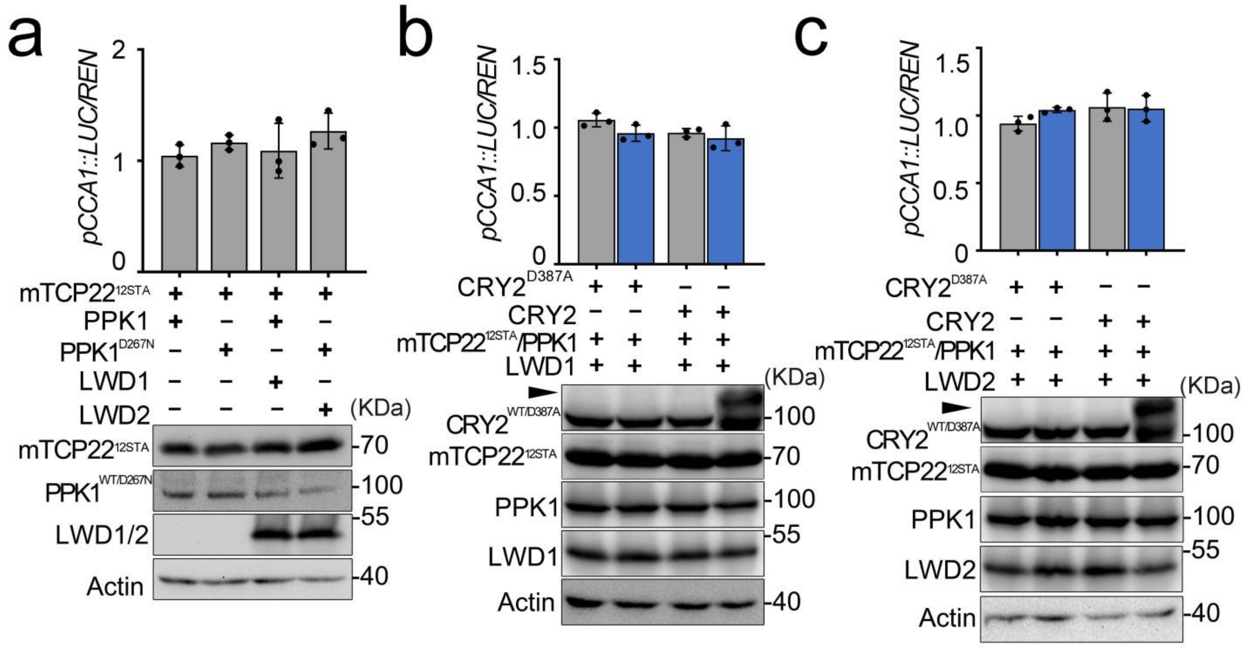

Supplementary Fig. 13 mTCP22^{12STA} failing to activate the expression of *CCA1*.

a, PPK1, LWD1 or LWD2 failed to promote the expression of *CCA1* with mTCP22^{12STA} as shown by the dual luciferase assay. *pCCA1::LUC* was co-expressed with the indicated proteins in HEK-293T cells. The relative luciferase (LUC) signal was normalized to the Renilla luciferase (REN) signal. Data are presented as mean ± SD (n = 3 independent assays). The expression levels of each protein in each sample

were estimated by immunoblot. **b**, **c**, CRY2 or CRY2^{D387A} could not enhance the transcriptional activity of mutant-TCP22^{12STA}-LWD1 nor mutant-TCP22^{12STA}-LWD2. (**b**) mutant-TCP22^{12STA}-LWD1, (**c**) mutant-TCP22^{12STA}-LWD2. Samples were kept in the dark (Grey columns) or exposed to blue light (Blue columns, 30 μmol m^{-2} s^{-1}) for 3 h before being lysed. Data are presented as mean ± SD (n = 3 independent assays).

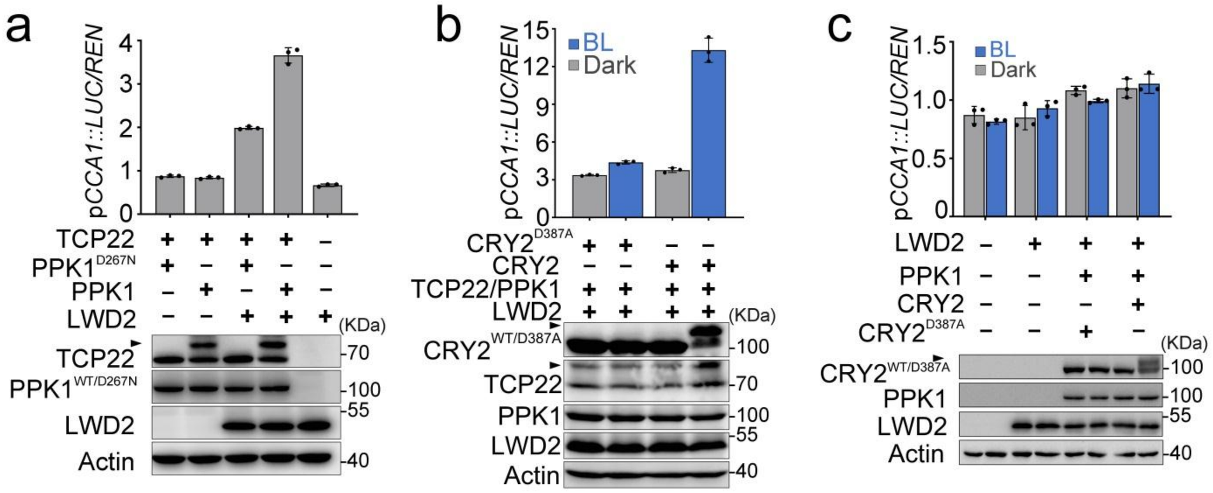

Supplementary Fig. 14 The coordination of CRY2, TCP22, LWD2 and PPK1 to activate *CCA1*.

a, TCP22 needed to recruit LWD2 to promote the expression of *CCA1* as shown by the dual luciferase assay. PPK1^{D267N} was the negative control of this assay. *pCCA1::LUC* was co-expressed with the indicated proteins in HEK-293T cells. The relative luciferase (LUC) signal was normalized to the Renilla luciferase (REN) signal. Data are presented as mean ± SD (n = 3 independent assays). Expression levels of each protein in each sample were estimated by immunoblot. **b**, CRY2 recruited PPK1 to enhance the transcriptional activity of TCP22-LWD2 complex for promotion of *CCA1* expression as shown by the dual luciferase assay. HEK-293T cells co-expressing with the indicated proteins were irradiated by blue light (BL, 30 μmol m^{-2} s^{-1}) or kept in dark before the measurement of LUC/REN signal. Data are presented as mean ± SD (n = 3 independent assays). Expression levels of each protein in each sample were estimated by immunoblot. Arrowhead indicated phosphorylated

TCP22 and CRY2. **c**, CRY2, LWD2, PPK1 could not activate the expression of *CCA1* in the absence of TCP22 under blue light or dark treatment as shown by the dual luciferase assay. The experiment and the condition were the same as (**b)**, except HEK-293T expressing different proteins. Data are presented as mean ± SD (n = 3 independent assays). Expression levels of each protein in each sample were estimated by immunoblot.

Supplementary Table 1. Primers used in this study

Primers for constructs used for expressing recombinant proteins in HEK293T Cells

Primer name	Primers
Myc to pCI(neo) F	5'-CTAGCCTCGAGAATTCATGGGGTTAATTAACGGTGA-3'
Myc to pCI(neo) R	5'-TACCACGCGTGAATTCGCTACCGTTCAAGTCTTCC-3'
Flag to pCI(neo) F	5'-CTAGCCTCGAGAATTCATGACTGATTACAAGGATG-3'
Flag to pCI(neo) R	5'-TACCACGCGTGAATTCACCTCCACCACCTCCTCCCT-3'
GFP to pCI(neo) F	5'-CTAGCCTCGAGAATTCATGGTGAGCAAGGGCGCCGA-3'
GFP to pCI(neo) R	5'-TACCACGCGTGAATTCCTTGTACAGCTCATCCATGC-3'
CRY2 to pCI(neo) F	5'-GCGTGGTACCTCTAGAATGAAGATGGACAAAAAGAC-3'
CRY2 to pCI(neo) R	5'-GAAGCGGCCGCCCGGGTCATTTGCAACCATTTTTTC-3'
PPK1 to pCI(neo) F	5'-GCGTGGTACCTCTAGAATGCCGGAGCTTCGCCGTGG-3'
PPK1 to pCI(neo) R	5'-GAAGCGGCCGCCCGGGTCAAGATACAGTTCGGCCAT-3'
LWD1 to pCI(neo) F	5'-GCGTGGTACCTCTAGAATGGGAACGAGCAGCGATCC-3'
LWD1 to pCI(neo) R	5'-GAAGCGGCCGCCCGGGTCAAACCCTGAGAATTTGCA-3'
LWD2 to pCI(neo) F	5'-GCGTGGTACCTCTAGAATGGTTACGAGCAGCGATCA-3'
LWD2 to pCI(neo) R	5'-GAAGCGGCCGCCCGGGTCAGACCCGGAGAATCTGCA-3'
TCP22 to pCI(neo) F	5'-GCGTGGTACCTCTAGAATGAATCAGAATTCCTCTGT-3'
TCP22 to pCI(neo) R	5'-GAAGCGGCCGCCCGGGTCACTTTTTGTCATCACCAC-3'

Primers for constructs used for dual luciferase assay in HEK-293T

Primer name	Primers
pNL2.2 F	5'-TTGTGTAGAGGAGCTTAGTGGCTAGCATGGAAGACG-3'
pNL2.2 R	5'-GAAACATCAGTGGACCGAGCTCAGGTACCGGCCAGT-3'
pNL2.2-CCA1(635) F	5'-GCCGGTACCTGAGCTCGGTCCACTGATGTTTCTAGT-3'
pNL2.2-CCA1(635) R	5'-GTCTTCCATGCTAGCCACTAAGCTCCTCTACACAAC-3'

Primers for constructs used for plant transformation

Primer name	Primers
TCP22 to pDT1 F	5'-CGACCTCACCCCCGGGATGAATCAGAATTCCTCTGT-3'
TCP22 to pDT1 R	5'-GCTTGGATCCCCCGGGTCACTTTTTGTCATCACCAC-3'
p3301-CCA1(984)-F	5'-TTACGAATTCGAGCTCGTCTCTGGTCTTTTTTAGCT-3'
p3301-CCA1(984)-R	5'-TTTGGCGTCTTCCATCACTAAGCTCCTCTACACAAC-3'
p3301-LUC-F	5'-TAGAGGAGCTTAGTGATGGAAGACGCCAAAAACAT-3'
p3301-LUC-R	5'-GAGAAAGCTTGGATCCCTACTTTCCGCCCTTCTTGG-3'
p3301-TCP22Pro F	5'-TTACGAATTCGAGCTCATGGGTTTAGAAGGAGTCAT-3'
p3301-TCP22Pro R	5'-CGTTAATTAACCCCATCTTCAAATCCGTAAAAAGAT-3'
p3301-Myc-TCP22 F	5'-TTTTACGGATTTGAAGATGGGGTTAATTAACGGTGA-3'
p3301-Myc-TCP22 R	5'-GAGAAAGCTTGGATCCTCACTTTTTGTCATCACCAC-3'

Primers for constructs used for protein expression and purification

Primer name	Primers
pCI(neo) his-LWD1 F	5'-TAGCCTCGAGAATTCATGCATCATCATCATCATCACATGGGAACGAGCAGCGATCC-3'
pCI(neo) his-LWD1 R	5'-AGCATTAACCCTCACTAAAGGGAAGCGGCCGCCCGGGTCAAACCCTGAGAATTTGCA-3'
pCI(neo) his-PPK1 F	5'-CTAGCCTCGAGAATTCATGCATCATCATCATCATCACATGCCGGAGCTTCGCCGTGG-3'
pCI(neo) his-PPK1 R	5'-AGCATTAACCCTCACTAAAGGGAAGCGGCCGCCCGGGTCAAGATACAGTTCGGCCAT-3'
PET His-TEV TCP22 F	5'-CTTCCAAGGGGGATCCATGAATCAGAATTCCTCTGT-3'
PET His-TEV TCP22 R	5'-CGACGATATCGAATTCTCACTTTTTGTCATCACCAC-3'

Primers for constructs used for yeast two-hybrid assays

Primer name	Primers
TCP22 AD F	5'-GGAGGCCAGTGAATTCATGAATCAGAATTCCTCTG-3'
TCP22 AD R	5'-CGAGCTCGATGGATCCTCACTTTTTGTCATCACCAC-3'
CRY2 BD F	5'-TGTATCGCCGGAATTCATGAAGATGGACAAAAAGAC-3'
CRY2 BD R	5'-GCAGGTCGACGGATCCTCATTTGCAACCATTTTTTC-3'
PPK1 BD F	5'-TGACTGTATCGCCGGAATTCATGCCGGAGCTTCGCC-3'
PPK1 BD R	5'-GCAGGTCGACGGATCCTCAAGATACAGTTCGGCCAT-3'
PPK2 BD F	5'-TGTATCGCCGGAATTCATGCCAGAGTTAAGAAGTGG-3'
PPK2 BD R	5'-CTGCAGGTCGACGGATCCTCAGCAAACTGTCCTCCC-3'
PPK3 BD F	5'-TGTATCGCCGGAATTCATGCCAGAGTTAAGAAGTGG-3'
PPK3 BD R	5'-GCAGGTCGACGGATCCTCAGCAAACTGTCCGACCAT-3'
PPK4 BD F	5'-TGTATCGCCGGAATTCATGCCTGAGCTGCGTAGC-3'
PPK4 BD R	5'-GCAGGTCGACGGATCCTCATGACACAGTTCGACCAT-3'

Primers for constructs used for chromatin immunoprecipitation assays

Primer name	Primers
pCCA1-a-F	5'-TGTCAAAGTGTTGTAAATTCCTCAAGA -3'
pCCA1-a-*R*	5'-GCATGAAGGGTAGAAGACTAAATGG -3'
pCCA1-b-F	5'-TCGACAAACTGGTGGGAGAG-3'
pCCA1-b-R	5'-TCCGGGACTACCTGAAAGGTT-3'
UBC21-F	5'-TTCAAATGGACCGCTCTTATCA-3'
UBC21-R	5'-AAACACCGCCTTCGTAAGGA-3'

Primers for constructs used for protoplast transformation

Primer name	Primers
105 TCP22 F	5'-CAAGGCCGGCGGATCCATGAATCAGAATTCCTCTGT-3'
105 TCP22 R	5'-GCAGGTCGACTCTAGATCACTTTTTGTCATCACCAC-3'

103 TCP22 F	5'-AGGTACCCGGGGATCCATGAATCAGAATTCCTCTGT-3'
103 TCP22 R	5'-CGCCGTCGACTCTAGATCACTTTTTGTCATCACCAC-3'
105 CRY2 F	5'-TACAATTACAGGTACCATGAAGATGGACAAAAAGAC-3'
105 CRY2 R	5'-CGCCGTCGACTCTAGATTTGCAACCATTTTTTCCCA-3'
103 CRY2 F	5'-AGGTACCCGGGGATCCATGAAGATGGACAAAAAGAC-3'
103 CRY2 R	5'-CGCCGTCGACTCTAGATCATTTGCAACCATTTTTTC-3'
105 PPK1 F	5'-TACAATTACAGGTACCATGCCGGAGCTTCGCCGTGG-3'
105 PPK1 R	5'-CGCCGTCGACTCTAGATCAAGATACAGTTCGGCCAT-3'
105 PPK2 F	5'-TACAATTACAGGTACCATGCCAGAGTTAAGAAGTGG-3'
105 PPK2 R	5'-CGCCGTCGACTCTAGATCAGCAAACTGTCCTCCCA-3'
105 PPK3 F	5'-TACAATTACAGGTACCATGCCAGAGTTAAGAAGTG-3'
105 PPK3 R	5'-CGCCGTCGACTCTAGATCAGCAAACTGTCCGACCA-3'
105 PPK4 F	5'-TACAATTACAGGTACCATGCCTGAGCTGCGTAGCAA-3'
105 PPK4 R	5'-CGCCGTCGACTCTAGATCATGACACAGTTCGACCA-3'
mCherry F	5'-TACAATTACAGGTACCATGGTGAGCAAGGGCGAGGA-3'
mCherry R	5'-ACCCGGAGGTGGTCCGGATCCCTTGTACAGCTCGTCCAT-3'
mCherry PPK1 F	5'-CGAGCTGTACAAGGGATCCGGACCACCTCCGGGTATGGA CAGGGAGGATAACCT-3
mCherry PPK1 R	5'-CTGCAGGTCGACTCTAGATTAATTTTCTCCTTCAAGACCT T-3'

Primers for constructs used for obtaining *mTCP22*^{12STA}

Primer name	Primers
mTCP22 pCI F	5'-GCGTGGTACCTCTAGAATGAATCAGAATTCCTCTG-3'
mTCP22 R	5'-CGGCCGCAGCGGCTGCCGCGGCTGCAGC-3'
mTCP22 F	5'-GCAGCCGCTGCGGCCGCAGCGGCTGGTGCTGCTCTT-3'
mTCP22 pCI R	5'-GAAGCGGCCGCCCGGGTCACTTTTTGTCATCACCA-3'
