# Supplementary material for: Arabidopsis cryptochrome 2 forms photobodies with TCP22 under blue light and regulates the circadian clock
Source: Nat Commun. 2022 May 12;13:2631. doi: 10.1038/s41467-022-30231-9 (PMC9098493; doi:10.1038/s41467-022-30231-9)
Supplement: Supplementary file 3 — Reporting Summary [file 41467_2022_30231_MOESM3_ESM.pdf]

nature research

Corresponding author(s): Zecheng Zuo

Last updated by author(s): Apr 4, 2022

Reporting Summary

Nature Research wishes to improve the reproducibility of the work that we publish. This form provides structure for consistency and transparency in reporting. For further information on Nature Research policies, see our Editorial Policies and the Editorial Policy Checklist.

Statistics

For all statistical analyses, confirm that the following items are present in the figure legend, table legend, main text, or Methods section.

n/a	Confirmed	
☐	☒	The exact sample size (*n*) for each experimental group/condition, given as a discrete number and unit of measurement
☐	☒	A statement on whether measurements were taken from distinct samples or whether the same sample was measured repeatedly
☐	☒	The statistical test(s) used AND whether they are one- or two-sided<br>*Only common tests should be described solely by name; describe more complex techniques in the Methods section.*
☒	☐	A description of all covariates tested
☐	☒	A description of any assumptions or corrections, such as tests of normality and adjustment for multiple comparisons
☐	☒	A full description of the statistical parameters including central tendency (e.g. means) or other basic estimates (e.g. regression coefficient) AND variation (e.g. standard deviation) or associated estimates of uncertainty (e.g. confidence intervals)
☐	☒	For null hypothesis testing, the test statistic (e.g. *F*, *t*, *r*) with confidence intervals, effect sizes, degrees of freedom and *P* value noted<br>*Give P values as exact values whenever suitable.*
☒	☐	For Bayesian analysis, information on the choice of priors and Markov chain Monte Carlo settings
☒	☐	For hierarchical and complex designs, identification of the appropriate level for tests and full reporting of outcomes
☒	☐	Estimates of effect sizes (e.g. Cohen's *d*, Pearson's *r*), indicating how they were calculated

Our web collection on statistics for biologists contains articles on many of the points above.

Software and code

Policy information about availability of computer code

Data collection	LAS X(Ver 3.4.2.18368) in confocal microscope imaging system Leica TCS SP8X was used to capture images. GelCapture (Version 7.0.18,DNR Bioimagining systems) were used to expose and capture image in western-blot assays. MikroWin (Ver 4.14) was used in Bioluminescence assay.
Data analysis	LAS X(VER 3.4.2.18368) was used to analyze the confocal images and FRAP images. Image J (1.52v) was used to analyze the particle information (size, number, circularity, partition ratio). EXCEL 2016 was used in two-sided student test and QPCR data analysis. GraphPad Prism 8.0.2 was used in SD calculation and forming graph. Adobe Photoshop CC 2017 was used to cut the images from western-blot and confocal microscope. Skyline daily (version 3.5),Thermo Proteome Discoverer (2.2.0.388), Maxquant (ver1.6.2.10) were used in Mass Spectrometry analysis.

For manuscripts utilizing custom algorithms or software that are central to the research but not yet described in published literature, software must be made available to editors and reviewers. We strongly encourage code deposition in a community repository (e.g. GitHub). See the Nature Research guidelines for submitting code & software for further information.

Data

Policy information about availability of data

All manuscripts must include a data availability statement. This statement should provide the following information, where applicable:

- Accession codes, unique identifiers, or web links for publicly available datasets
- A list of figures that have associated raw data
- A description of any restrictions on data availability

The source data for Figs. 1-5, Supplementary Figs. 3-14 are provided with this paper as a Source Data file. Other data and materials of this study are available from

the corresponding author upon reasonable request. The mass spectrometry data for CRY2-interacting proteomics and phosphorylation sites in TCP22 were have been deposited to the ProteomeXchange Consortium via the PRIDE partner repository with the dataset identifiers PXD032848 and PXD032849, respectively.

Field-specific reporting

Please select the one below that is the best fit for your research. If you are not sure, read the appropriate sections before making your selection.

☒ Life sciences ☐ Behavioural & social sciences ☐ Ecological, evolutionary & environmental sciences

For a reference copy of the document with all sections, see nature.com/documents/nr-reporting-summary-flat.pdf

Life sciences study design

All studies must disclose on these points even when the disclosure is negative.

Sample size	No statistical methods were used to predetermine sample size. For the circadian clock detection, seedlings (n>30) were cultured in the 96-well plates and more than 10 replications were used to detect the circadian rhythm. For each BiFC assay, more than 5 independent plants of same phenotype were used to isolate the protoplasts. We used 3 biological replicates for all bioluminescence test in HEK-293T cells and 2 biological replicates for the ChIP-qPCR.
Data exclusions	No data were excluded
Replication	The attempts of each replication were successful. Most of experiments in this paper was replicated on separate days and sharing the similar results.
Randomization	All samples were collected randomly into experimental groups
Blinding	Blinding was not performed for the experiments in this paper

Reporting for specific materials, systems and methods

We require information from authors about some types of materials, experimental systems and methods used in many studies. Here, indicate whether each material, system or method listed is relevant to your study. If you are not sure if a list item applies to your research, read the appropriate section before selecting a response.

Materials & experimental systems

n/a	Involved in the study	
☐	☒	Antibodies
☐	☒	Eukaryotic cell lines
☒	☐	Palaeontology and archaeology
☒	☐	Animals and other organisms
☒	☐	Human research participants
☒	☐	Clinical data
☒	☐	Dual use research of concern

Methods

n/a	Involved in the study	
☒	☐	ChIP-seq
☒	☐	Flow cytometry
☒	☐	MRI-based neuroimaging

Antibodies

Antibodies used

Anti-GFP (Green Fluorescent Protein) pAb (598, Rabbit IgG, 1:3000 dilution for Western-Blot, MBL, Lot081)
Anti-Myc-tag mAb (M192-3, Mouse IgG2bκ, 1:3000 dilution for Western-Blot, 1:100 dilution for immunostaining, MBL, Lot.006) Anti-DDDDK-tag mAb (M185-3L, Mouse IgG2aκ, 1:3000 dilution for Western-Blot, MBL, Lot.007)
Anti-β-Actin mAb (M177-3, Mouse IgG1 κ, 1:3000 dilution for Western-Blot, MBL, Lot.003)
Anti-Luciferase pAb (PM016, Rabbit IgG, 1:3000 dilution for Western-Blot, MBL, Lot.014)
Anti-HSP [illegible]AbM51099-31-PU, Mouse IgG2a, 1:3000 dilution for Western-Blot, Beijing Protein Innovation[illegible]
Anti-CRY2 antibody is prepared in our lab (Ref: Science. 1988. 279: 1360-1363, 1:3000 dilution for Western-Blot, 1:100 dilution for immunostaining)
CY3 conjugated goat anti-rabbit IgG (A0516, Rabbit IgG, 1:500 dilution for immunostaining, Beyotime)
Alexa Fluor 488 conjugated goat anti-mouse IgG (A0428, Mouse IgG, 1:500 dilution for immunostaining, Beyotime)

Validation

All antibody used in this study were certified and validated by manufactures and vendors
The details about Anti-GFP (Green Fluorescent Protein) pAb is in http://www.mbl-chinawide.cn/search-details2?id=85&table=RuoAntibody
The details about Anti-Myc-tag mAb is in http://www.mbl-chinawide.cn/search-details2?id=989&table=RuoAntibody
The details about Anti-DDDDK-tag mAb is in http://www.mbl-chinawide.cn/search-details2?id=833&table=RuoAntibody
The details about Anti-β-Actin mAb is in http://www.mbl-chinawide.cn/search-details2?id=20887&table=RuoAntibody
The details about Anti-Luciferase pAb is in http://www.mbl-chinawide.cn/search-details2?id=1576&table=RuoAntibody

The details about Anti-HSP is in http://www.proteomics.org.cn/product/202.html

Eukaryotic cell lines

Policy information about cell lines

Cell line source(s)	HEK-293T cells were obtained from ATCC(ATCC®CRL-11268TM)
Authentication	The cell line used in this paper from ATCC were not specifically re-authenticated.
Mycoplasma contamination	The HEK293T cell line used in this study was not specifically tested for Mycoplasma contamination. However, no abnormalities of cultured cells have been observed, such as slowed cell growth and interfered cell attachment.
Commonly misidentified lines (See ICLAC register)	No commonly misidentified lines were used in this paper.
